# A new species of *Phosocephala* Townsend, 1908 (Diptera: Tachinidae) from Area de Conservación Guanacaste in northwestern Costa Rica

**DOI:** 10.3897/BDJ.4.e7863

**Published:** 2016-04-25

**Authors:** AJ Fleming, D. Monty Wood, M. Alex Smith, Daniel H Janzen, Winnie Hallwachs, Tanya Dapkey

**Affiliations:** ‡ Agriculture Agri-Food Canada, Ottawa, Canada; § Department of Integrative Biology and the Biodiversity Institute of Ontario, Guelph, Canada; | Department of Biology, University of Pennsylvania, Philadelphia, United States of America

**Keywords:** Tachininae, Tachinini, tropical rain forest, tropical dry forest, parasitoid flies, host specificity, Erebidae, Nolidae

## Abstract

**Background:**

We describe a new species of *Phosocephala* Townsend, and provide a new collection record, and description of the previously unknown male, of *Phosocephala metallica* Townsend, from Area de Conservación Guanacaste (ACG), northwestern Costa Rica. All ACG specimens were reared from wild-caught Lepidoptera larvae (Lepidoptera: Erebidae, Nolidae). We provide a concise description of both species using morphology, life history, molecular data, and photographic documentation. The new species is authored and described by Fleming and Wood.

**New information:**

*Phosocephala alexanderi* **sp. n.**

## Introduction

The genus *Phosocephala* Townsend, 1908 (Tachininae: Tachinini) belongs to the tribe Tachinini in the subfamily Tachininae. Tachininae parasitoids are ovo-larviparous, laying their eggs on or near the host. Once the eggs havehatched, the first instar larvae seek out the host and burrow through its integument (Stireman et al. 2006). The genus was previously known only from the holotype, originally described by Townsend (1908). Townsend erected the new genus based on the single female specimen collected from the rain forest of Tucurrique, Costa Rica, during an expedition by Messrs. Schild and Burgdorf, which he described as *Phosocephala metallica* Townsend.

The present study is part of a larger group of studies documenting the tachinid species living within the 120,000 km^2^ terrestrial part of Area de Conservación Guanacaste (http://www.acguanacaste.ac.cr), and name the undescribed species discovered during the project. To date, two species of *Phosocephala* have been reared from Area de Conservación Guanacaste. Using CO1 (cox1 or cytochrome oxidase 1) gene sequences or “DNA barcodes”, life history, and morphological differences as diagnostic characters, we describe a new species, and provide a redescription, the description of the male, and a new record for *P. metallica* Townsend.

## Materials and methods

**Study area and rearing intensity**

All flies and rearing information described here were collected within the 35+ year–old ongoing inventory of the caterpillars, their food plants, and their parasitoids, across the three major ecosystems of the terrestrial portion of Area de Conservación Guanacaste (ACG) in northwestern Costa Rica (Smith et al. 2005, Smith et al. 2006, Smith et al. 2007, Smith et al. 2008, Smith et al. 2009, Smith et al. 2012, Janzen et al. 2009, Janzen and Hallwachs 2011, Rodriguez et al. 2012, Fleming et al. 2014a, Fleming et al. 2014b, Fleming et al. 2015c, Fleming et al. 2015d). The parasitoid rearing methods are described by Janzen et al. (2009) and at http://janzen.bio.upenn.edu/caterpillars/methodology/how/parasitoid_husbandry.htm.

This inventory has reared more than 600,000 wild-caught caterpillars since (1978). All frequencies of parasitization reported here need to be considered against this background inventory (Janzen et al. 2009, Janzen and Hallwachs 2011, Fernandez-Triana et al. 2014).

The individual and comparative details of the parasitization ecology of these flies will be treated in later papers, once the overall knowledge of the caterpillar-attacking tachinids found in ACG is more complete (Smith et al. 2006, Smith et al. 2007, Fleming et al. 2014a, Fleming et al. 2014b, Fleming et al. 2015a, Fleming et al. 2015b, Fleming et al. 2015c, Fleming et al. 2015d).

**Imaging**

Habitus and terminalia photographs were taken using the methods outlined in Fleming et al. (2014a). Raw image files were first processed with Adobe Photoshop CS6, then digitally stacked to produce a final composite image using Zerene Stacker Software v1.04.

Adult flies were dissected following standard practice (O’Hara 1983, Fleming et al. 2014). The morphological terminology follows Cumming and Wood (2009).

**Acronyms for depositories**

CNC - Canadian National Collection of Insects, Arachnids and Nematodes, Ottawa, Canada

MNCR - Museo Nacional de Costa Rica (formerly Instituto Naciónal de Biodiversidad), San José, Costa Rica

USNM - National Museum of Natural History (formerly United States National Museum), Washington, D.C., U.S.A

**Voucher specimen management**

The management of voucher specimens has been detailed in previous papers in this series (Fleming et al. 2014a, Fleming et al. 2014b, Fleming et al. 2015a, Fleming et al. 2015b, Fleming et al. 2015c, Fleming et al. 2015d). In brief, caterpillars reared from the ACG efforts receive a unique voucher code in the format yy–SRNP–xxxxx. Parasitoids emerging from a caterpillar receive the same voucher code; when/ifthey are later individually processed for DNA barcoding, they receive a second, unique voucher code in the format DHJPARxxxxxxx. The associated data for each voucher code are available at: http://janzen.bio.upenn.edu/caterpillars/database.lasso.

All DHJPARxxxxxxx-coded tachinids had one leg removed for DNA barcoding and couriered to the Biodiversity Institute of Ontario (BIO) in Guelph. All associated data and successful barcodes are permanently and publicly deposited in the Barcode of Life Data System (BOLD) (Ratnasingham and Hebert 2007), and a select set of these data subsequently migrated to GenBank. Each barcoded specimen also received accession numbers from the Barcode of Life Data System (BOLD) and GenBank, respectively. Because the inventory is continually adding new specimens, these can be found by searching for the genus *Phosocephala* in BOLD.

All inventoried specimens discussed herein were collected under Costa Rican government research permits issued to DHJ, and the Tachinidae samples were exported under permit by DHJ from Costa Rica to their final depository in the CNC. Tachinid identifications for the inventory were done by DHJ in coordination with a) visual inspection by AJF and DMW, b) DNA barcoding by MAS and the Biodiversity Institute of Ontario, and c) databasing and association with host caterpillars by DHJ and WH via the inventory itself.

The dates of capture cited for each fly are the dates of eclosion of the fly, and not the date of capture of the caterpillar. The fly eclosion date is much more representative of the time when that species is on the wing than is the time of capture of the parasitized caterpillar. The “collector” is the parataxonomist who found the caterpillar, rather than the person who later retrieved the newly eclosed fly and processed it by freezing, pinning, labeling and oven–drying. The biology and parasitization rates of individual tachinid speciesecies will be the subject of later papers. The holotype of the new species is deposited in the Diptera collection of the CNC.

**Interim names for undescribed host species**

Names of undescribed host species follow a standardized, interim naming system used for taxonomic units considered as distinct species and identified by DNA barcodes. The interim names are given in the format "*Eois* Janzen52", where the species epithet is composed of the name of the taxonomist who identified the species and a number. This prevents confusion with already described species while maintaining traceability of each undescribed species within the ACG project.

**DNA barcoding**

The standard DNA barcode region from the 5’ end of the mitochondrial cytochrome *c* oxidase I (CO1) gene was obtained for all five ACG inventory specimens presented here using DNA extractions obtained from single legs, using a glass fiber protocol (Ivanova et al. 2006). Total genomic DNA was re-suspended in 30 μl of dH_2_O, and the 658-bp barcode region near the 5’ terminus of the CO1 gene was amplified using standard primers (LepF1–LepR1) and following established protocols (Smith et al. 2006, Smith et al. 2007, Smith et al. 2008). All information pertaining to the sequences associated with each individual specimen (including GenBank and BOLD accession) can be retrieved from the Barcode of Life Data System (BOLD) (Ratnasingham and Hebert 2007) via the publicly available dataset: http://dx.doi.org/10.5883/DS-ASPHOSC

## Taxon treatments

### Phosocephala

Townsend, 1908

Phosocephala ***Phosocephala* Townsend, 1908**
Phosocephala Townsend, 1908: 69. Type species: *Phosocephala metallica* Townsend, 1908, by original designation.
Phosocephala Phosocephala metallica Townsend, 1908Townsend 1908: 69.

#### Description

*Phosocephala* belongs to the tribe Tachinini. The tribe Tachinini is defined by the presence ofthe following characters: first flagellomere elongate, bean-shaped; prosternum bare; upper side of calypter bare; first postsutural supra-alar bristle at least as long and stout as first postsutural dorsocentral bristle; hind coxa setose.

The following redescription applies to both males and females, which differ only very slightly; any differences between the sexes are noted. **Head:** bright yellow to dark orange, about as wide as thorax; width of frons greater than 1/2 of head width; head flattened laterally, approximately 2x longer than wide; frontal bristles not descending beyond middle of pedicel, upper two pairs of frontal bristles strong and reclinate; fronto-orbital plate with 2 pairs of strong proclinate orbital bristles; 2 pairs of reclinate vertical bristles; ocellar bristles well developed, proclinate and strongly divergent; fronto-orbital plate haired; frons concolorous with parafrontal, only differentiated by its lack of hairs; parafacial densely haired, though these hairs are not obvious unless viewed under varying angles of light; facial carina flat or absent; antennal grooves only slightly distinguishable; palpus entirely absent; eyes bare; first flagellomere approximately 2x as long as pedicel, brilliant orange, with hints of darkbrown; arista bare; gena from 0.3x to 0.5x height of eye (longer in females), and densely haired with short black to yellow-orange hairs. **Thorax:** thorax shiny metallic black over 90% of its surface, with a dense shiny golden pollinosity along the anterior edge; 4 gray pruinose thoracic vittae only visible under certain angles of light; prosternum bare; proepisternum bare; postpronotum translucent yellow, contrasting with remainder of scutellum, which is a metallic black; 5–7 meral bristles; anepimeron with 2 strong bristles; katepisternum bearing 3–4 bristles; anepisternum with 7 bristles along posterior fringe; 3 postsutural supra-alar bristles; 3 dorsocentral bristles; 2–3 pairs of lateral scutellar bristles and 1 pair of subapical scutellar bristles; apical scutellar bristles weak and convergent; scutellum with overall dark brown ground color, covered in a silver pollinosity. Wings distinctly smoky gray; wingvein R4+5 bearing only 2–3 bristles at base. **Legs:** entirely yellow, hirsute; posterior margin of metacoxa with 2–3 small, almost inconspicuous blond hairs; protibia with anterodorsal row of regularly spaced setae upper all equal in length, basal bristle 2X as long as the rest; claws and pulvilli short and thin, almost as long as 5th tarsomere. Mesotibia with 5 strong anterodorsal bristles, 2 anterior bristles almost 2/3 length of mesotibia, 2 posterodorsal bristles, and 1 ventral bristle. Metatibia with irregular row of anterodorsal setae, 4–5 posterodorsal and 2 anterior setae. **Abdomen:** subequal in length and width to thorax; mid-dorsal depression on T1+2 shallow and wide, extending almost to hind margin of tergite; anterior margins of tergites with narrow bands of thin silver pollinosity; median marginal bristles present on T3, and a row of marginal bristles on T4 and T5; discal bristles absent from all tergites; ground color of abdomen ranging from a deep blackish-burgundy, appearing shiny black when viewed from a distance, to a deep reddish-brown.

#### Species previously included

*metallica* Townsend, 1908: 72 (*Phosocephala*). Holotype female (USNM), by original designation [examined by DMW]. Type locality: Costa Rica, Tucurrique

### Phosocephala alexanderi

Fleming & Wood sp. n.

urn:lsid:zoobank.org:act:55186982-95E3-426F-A9AD-E4EAEDD18445

#### Materials

- **Type status:** Holotype. **Occurrence:** occurrenceDetails: http://janzen.sas.upenn.edu; catalogNumber: DHJPAR0048469; recordedBy: D.H. Janzen & W. Hallwachs, Freddy Quesada; individualID: DHJPAR0048469; individualCount: 1; sex: M; lifeStage: adult; preparations: pinned; otherCatalogNumbers: 12-SRNP-30536, ACGBA2011-12; **Taxon:** scientificName: Phosocephala alexanderi; phylum: Arthropoda; class: Insecta; order: Diptera; family: Tachinidae; genus: Phosocephala; specificEpithet: alexanderi; scientificNameAuthorship: Fleming & Wood, 2016; **Location:** continent: Central America; country: Costa Rica; countryCode: CR; stateProvince: Guanacaste; county: Sector Pitilla; locality: Area de Conservacion Guanacaste; verbatimLocality: Estacion Pitilla; verbatimElevation: 675; verbatimLatitude: 10.98931; verbatimLongitude: -85.42581; verbatimCoordinateSystem: Decimal; decimalLatitude: 10.98931; decimalLongitude: -85.42581; **Identification:** identifiedBy: AJ Fleming; dateIdentified: 2015; **Event:** samplingProtocol: Reared from an Erebidae moth larva, *Antiblemma* Poole12; verbatimEventDate: Apr-04-2012; **Record Level:** language: en; institutionCode: CNC; collectionCode: Insects; basisOfRecord: Pinned Specimen
- **Type status:** Paratype. **Occurrence:** occurrenceDetails: http://janzen.sas.upenn.edu; catalogNumber: DHJPAR0048468; recordedBy: D.H. Janzen & W. Hallwachs, Freddy Quesada; individualID: DHJPAR0048468; individualCount: 1; sex: F; lifeStage: adult; preparations: pinned; otherCatalogNumbers: 12-SRNP-30537, ACGBA2010-12; **Taxon:** scientificName: Phosocephala alexanderi; phylum: Arthropoda; class: Insecta; order: Diptera; family: Tachinidae; genus: Phosocephala; specificEpithet: alexanderi; scientificNameAuthorship: Fleming & Wood, 2016; **Location:** continent: Central America; country: Costa Rica; countryCode: CR; stateProvince: Guanacaste; county: Sector Pitilla; locality: Area de Conservacion Guanacaste; verbatimLocality: Estacion Pitilla; verbatimElevation: 675; verbatimLatitude: 10.98931; verbatimLongitude: -85.42581; verbatimCoordinateSystem: Decimal; decimalLatitude: 10.98931; decimalLongitude: -85.42581; **Identification:** identifiedBy: AJ Fleming; dateIdentified: 2015; **Event:** samplingProtocol: Reared from an Erebidae moth larva, *Antiblemma* Poole12; verbatimEventDate: Apr-06-2012; **Record Level:** language: en; institutionCode: CNC; collectionCode: Insects; basisOfRecord: Pinned Specimen

#### Description

Described from 1 male and 1 female. Length: 6mm.

**Head:** (Fig. 1b, e) bright yellow; ocellar bristles proclinate and strongly divergent; fronto-orbital plate haired, with yellow hairs insterspersed with black hairs; parafacial densely populated with short yellow hairs, not obvious unless viewed under varying angles of light; facial carina flat, nearly absent; antennal grooves only slightly distinguishable; facial ridge slightly darker than parafacial; first flagellomere brilliant orange, with hints of dark brown; gena 0.28x height of eye in male, 0.43x height of eye in female; gena densely haired, with short yellow-orange hairs, and with a small tuft of 4–5 short black hairs at base of eye. **Thorax:** (Fig. 1a, c, d, f) scutum gold pollinose presuturally, with 4 faintly visible vittae; postsuturally with gold only on the anterior margins along the corners, glabrous black over remainder including scutellum; thorax, laterally, of dark gray ground color with silver gray pollinosity giving it a silver sheen, with an overall dark appearance, and with long dark hairs; proepisternum, postpronotum, and proepimeron yellow; katepisternum, anepisternum, katepimeron and meron dark gray in ground color; katepisternum with 3–4 bristles. **Abdomen:** (Fig. 2a, c, d, f) ground color dark purplish black, base of T1+2 with silver pollinosity covering posterior 1/2 of underside of tergite, extending into anterior 1/4 of T3, anterior margins of tergites 3 and 4 bearing a narrow band of thin silver pollinosty visible laterally and ventrally, and also dorsally under certain angles of light. **Legs:** bright yellow, moderately covered with short black hairs. **Wings:** (Fig. 1a, d) smoky gray­­­­­­­­, bearing 4–5 short setulae at the base of R_4+5_. **Male terminalia:** not dissected so as not to damage the only available male specimen.

#### Diagnosis

The new species differs from *P. metallica* by its smaller size, the bright yellow head color, the black color of the posterior half of the thorax laterally, encompassing the katepisternum, anepisternum, katepimeron, and meron, and the long black hairs laterally on the thorax.

#### Etymology

*Phosocephala alexanderi* is dedicated to Mr. Alexander José Fleming of Ottawa, Canada, in recognition of the potential he has, as do all of the children of the world, to become the stewards and protectors of the biodiversity we describe here today.

#### Distribution

Costa Rica, ACG, Guanacaste, Estacion Pitilla, rain forest, 675m.

#### Ecology

Reared twice from larvae of *Antiblemma* Hübner belonging to the undescribed species *Antiblemma* Poole12 (Lepidoptera: Erebidae), which feeds on the leaves of *Conostegia xalapensis* (Melastomataceae).

### Phosocephala metallica

Townsend, 1908

#### Materials

- **Type status:** Holotype. **Occurrence:** occurrenceDetails: http://n2t.net/ark:/65665/39956764d-8fca-4c6c-a18b-a3946f68ea3c; catalogNumber: No.10902; recordedBy: D.M. Wood; individualID: No.10902; individualCount: 1; sex: F; lifeStage: adult; preparations: pinned; **Taxon:** scientificName: Phosocephala metallica; phylum: Arthropoda; class: Insecta; order: Diptera; family: Tachinidae; genus: Phosocephala; specificEpithet: metallica; scientificNameAuthorship: Townsend, 1908; **Location:** continent: Central America; country: Costa Rica; countryCode: CR; stateProvince: Cartago; county: Turrialba; locality: Tucurrique; **Event:** samplingProtocol: hand collected during field expedition of Schild and Burgdorf; verbatimEventDate: N/A; **Record Level:** language: en; institutionCode: USNM; collectionCode: Insects; basisOfRecord: Pinned Specimen
- **Type status:** Other material. **Occurrence:** occurrenceDetails: http://janzen.sas.upenn.edu; catalogNumber: DHJPAR0006933; recordedBy: D.H. Janzen & W. Hallwachs, Roster Moraga; individualID: DHJPAR0006933; individualCount: 1; sex: M; lifeStage: adult; preparations: pinned; otherCatalogNumbers: 06-SRNP-20280,BOLD:AAF5995,ASTAV175-06; **Taxon:** scientificName: Phosocephala metallica; phylum: Arthropoda; class: Insecta; order: Diptera; family: Tachinidae; genus: Phosocephala; specificEpithet: metallica; scientificNameAuthorship: Townsend, 1908; **Location:** continent: Central America; country: Costa Rica; countryCode: CR; stateProvince: Guanacaste; county: Sector Del Oro; locality: Area de Conservacion Guanacaste; verbatimLocality: Quebrada Trigal; verbatimElevation: 290; verbatimLatitude: 11.027; verbatimLongitude: -85.495; verbatimCoordinateSystem: Decimal; decimalLatitude: 11.027; decimalLongitude: -85.495; **Identification:** identifiedBy: AJ Fleming; dateIdentified: 2105; **Event:** samplingProtocol: Reared from a Nolidae moth larva, *Iscadia purpurascens*; verbatimEventDate: 15-Feb-2006; **Record Level:** language: en; institutionCode: CNC; collectionCode: Insects; basisOfRecord: Pinned Specimen
- **Type status:** Other material. **Occurrence:** occurrenceDetails: http://janzen.sas.upenn.edu; catalogNumber: DHJPAR0018275; recordedBy: D.H. Janzen & W. Hallwachs, Roster Moraga; individualID: DHJPAR0018275; individualCount: 1; sex: M; lifeStage: adult; preparations: pinned; otherCatalogNumbers: 03-SRNP-1672,BOLD:AAF5995,ASTAR985-07; **Taxon:** scientificName: Phosocephala metallica; phylum: Arthropoda; class: Insecta; order: Diptera; family: Tachinidae; genus: Phosocephala; specificEpithet: metallica; scientificNameAuthorship: Townsend, 1908; **Location:** continent: Central America; country: Costa Rica; countryCode: CR; stateProvince: Guanacaste; county: Sector Del Oro; locality: Area de Conservacion Guanacaste; verbatimLocality: Quebrada Trigal; verbatimElevation: 290; verbatimLatitude: 11.027; verbatimLongitude: -85.495; verbatimCoordinateSystem: Decimal; decimalLatitude: 11.027; decimalLongitude: -85.495; **Identification:** identifiedBy: AJ Fleming; dateIdentified: 2105; **Event:** samplingProtocol: Reared from a Nolidae moth larva, *Iscadia purpurascens*; verbatimEventDate: 18-Mar-2003; **Record Level:** language: en; institutionCode: CNC; collectionCode: Insects; basisOfRecord: Pinned Specimen
- **Type status:** Other material. **Occurrence:** occurrenceDetails: http://janzen.sas.upenn.edu; catalogNumber: DHJPAR0018276; recordedBy: D.H. Janzen & W. Hallwachs, Manuel Pereira; individualID: DHJPAR0018276; individualCount: 1; sex: F; lifeStage: adult; preparations: pinned; otherCatalogNumbers: 03-SRNP-18792,BOLD:AAF5995,ASTAR986-07; **Taxon:** scientificName: Phosocephala metallica; phylum: Arthropoda; class: Insecta; order: Diptera; family: Tachinidae; genus: Phosocephala; specificEpithet: metallica; scientificNameAuthorship: Townsend, 1908; **Location:** continent: Central America; country: Costa Rica; countryCode: CR; stateProvince: Guanacaste; county: Sector Del Oro; locality: Area de Conservacion Guanacaste; verbatimLocality: Quebrada Trigal; verbatimElevation: 290; verbatimLatitude: 11.027; verbatimLongitude: -85.495; verbatimCoordinateSystem: Decimal; decimalLatitude: 11.027; decimalLongitude: -85.495; **Identification:** identifiedBy: AJ Fleming; dateIdentified: 2105; **Event:** samplingProtocol: Reared from a Nolidae moth larva, *Iscadia purpurascens*; verbatimEventDate: 10-Sep-2003; **Record Level:** language: en; institutionCode: CNC; collectionCode: Insects; basisOfRecord: Pinned Specimen

#### Description

Described from 2 males and 2 females. Length: 8–9mm.

**Head:** (Fig. 2b, e) dark reddish-orange to dark yellow; ocellar bristles strongly lateraloclinate; fronto-orbital plate densely haired, with short dark hairs interspersed with yellow hairs; parafacial densely populated with short blond hairs, not obvious unless viewed under varying angles of light; facial carina flat to absent; antennal grooves almost indistinguishable; first flagellomere brilliant orange, with hints of dark brown; gena 0.3x height of eye in males, 0.36x height of eye in females; gena densely haired with short yellow-orange hairs, and with a small tuft of black hairs at base of eye, only apparent in the female. **Thorax:** (Fig. 2a, c, d, f) scutum gold-pollinose presuturally, with 4 faintly visible thoracic; scutum, postsuturally, with gold pollinosity only on the anterior corners, otherwise glabrous black, including scutellum; thorax, laterally, yellow in ground color, with long yellow hairs laterally, and a light gray pollinosity giving it a silver sheen; katepisternum with 3 bristles. **Abdomen:** (Fig. 2a, c, d, f) ground color deep shiny reddish-brown dorsally, changing to a lighter reddish-orange ventrally; T1+2 with silver pollinosity covering posterior 1/3 of underside of T1+2 extending into anterior 1/6 of T3; anterior margins of T3 and T4 with a narrow band of thin silver pollinosity, reaching the lateral and ventral sides of the tergites. **Legs:** bright yellow, with a dense covering of short black hairs. **Wings:** (Fig. 2a, d) smoky, translucent, bearing 4–5 short setulae at the base of R_4+5_. **Male terminalia:** (Fig. 3) sternite 5 with deeply excavated median cleft in a rounded V-shape; apical lobes squared at apex, densely covered in long thick bristles; cercus in dorsal view fused medially, apically pointed with a small bifurcation at tip, densely hirsute basally with numerous long bristles; surstylus divided into two fused processes, both with a strong inward curve; ventral process apically rounded, dorsal process apically hooked.

#### Diagnosis

*Phosocephala metallica* differs from *P. alexanderi* by its larger size, the dark orange head color, the yellow ground color of the thorax laterally, encompassing the katepisternum, anepisternum, katepimeron, and meron, and the conspicuous yellow hairs laterally on the thorax.

#### Distribution

Holotype: Costa Rica, Tucurrique (USNM). Other material: Costa Rica, ACG, Guanacaste, Quebrada Trigal, rain forest, 290m (CNC).

#### Ecology

This species was reared from three out of a total of 88 caterpillars of *Iscadia purpurascens* (Schaus, 1910) (Lepidoptera: Nolidae) bred during the project. The host species feeds on leaves of *Garcinia intermedia* (Clusiaceae).

## Analysis

The DNA barcode sequences recovered from the two species of ACG *Phosocephala* displayed the strong AT bias characteristic of insect mitochondrial DNA (mean percent GC content 30.33%, SE 0.1) and displayed no insertions or deletions. Within-species variation was low (mean distance of 0.39%) compared to between-species variation (mean distance 4.91%). All values of DNA barcode variation were calculated within BOLD and can be re-calculated in the future as more specimens or species are recovered from the ACG inventory and added to the DNA library. The neighbor-joining (NJ) tree (Saitou and Nei 1987) for the species of *Phosocephala* from ACG produced by BOLD is presented here as Suppl. material 1.

## Supplementary Material

Supplementary material 1 ACG Phosocephala NJ tree Data type: Neighbor-joining tree
File: oo_73368.pdf Fleming et al., 2016

XML Treatment for Phosocephala

XML Treatment for Phosocephala alexanderi

XML Treatment for Phosocephala metallica

## Figures and Tables

**Figure 1a.**
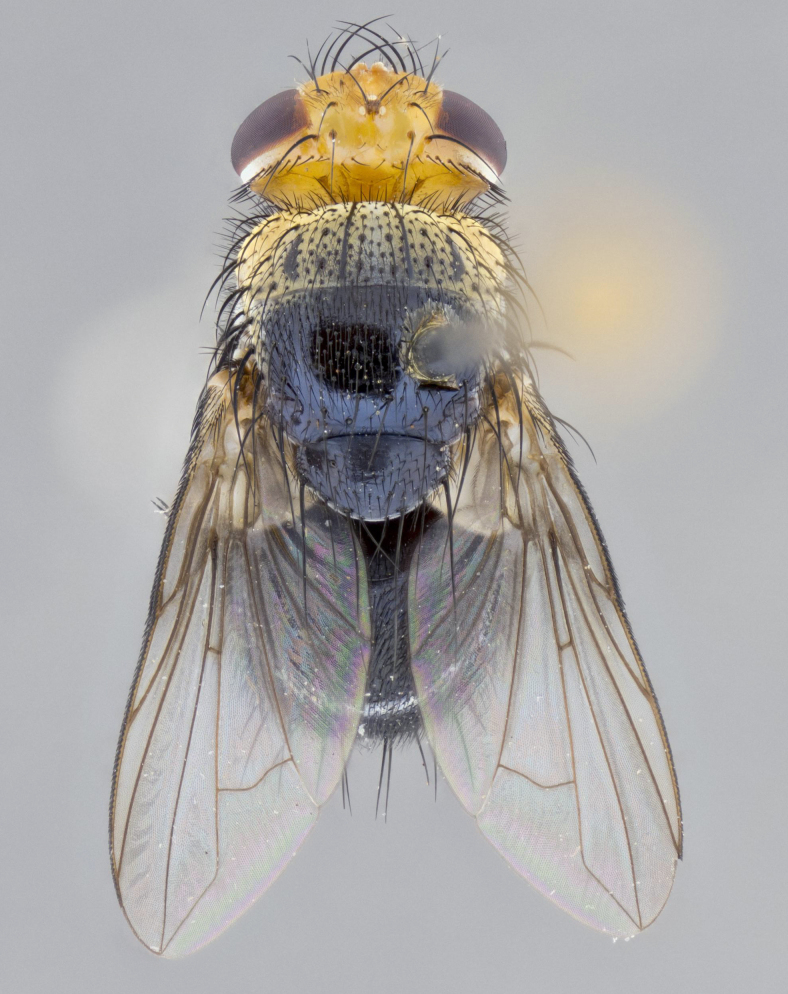
habitus in dorsal view

**Figure 1b.**
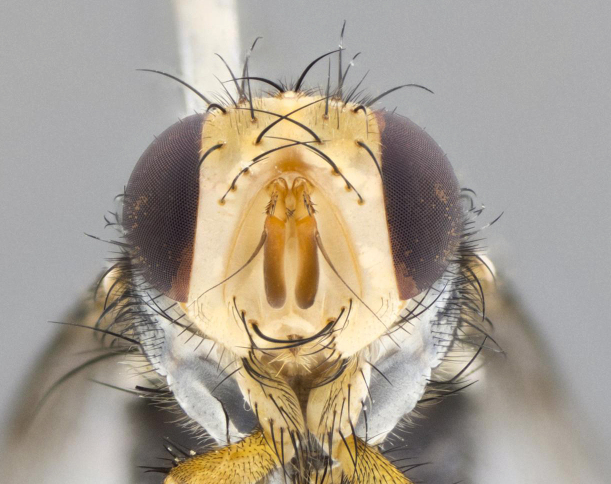
head in frontal view

**Figure 1c.**
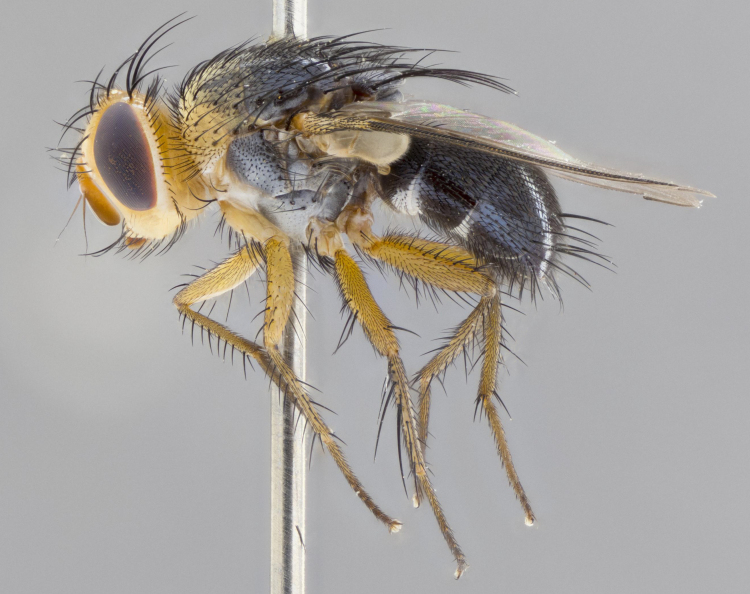
habitus in lateral view

**Figure 1d.**
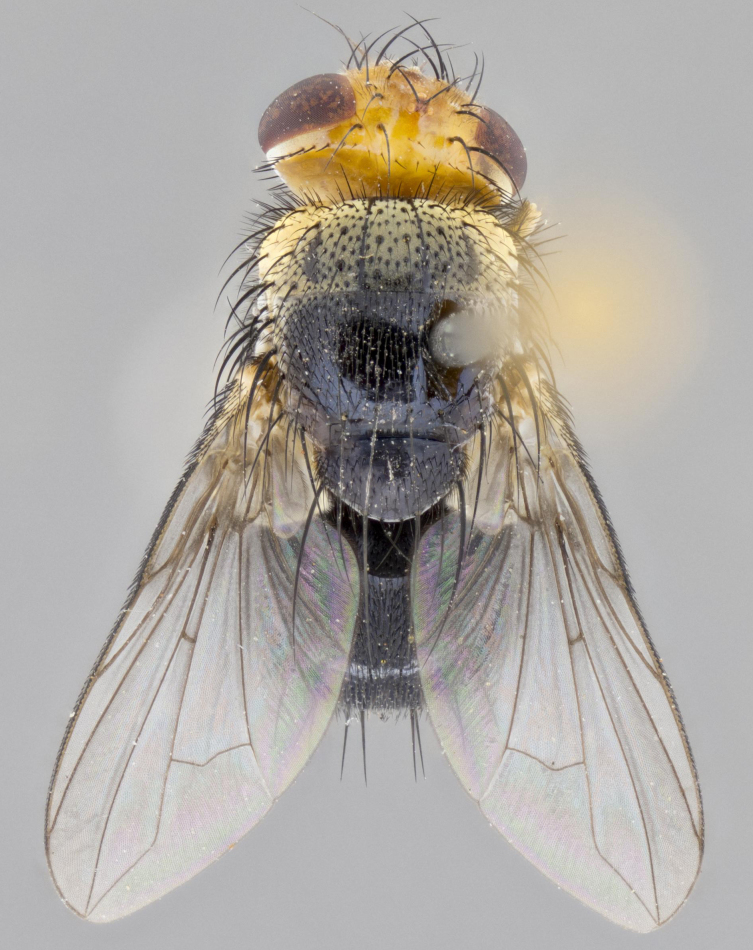
habitus in dorsal view

**Figure 1e.**
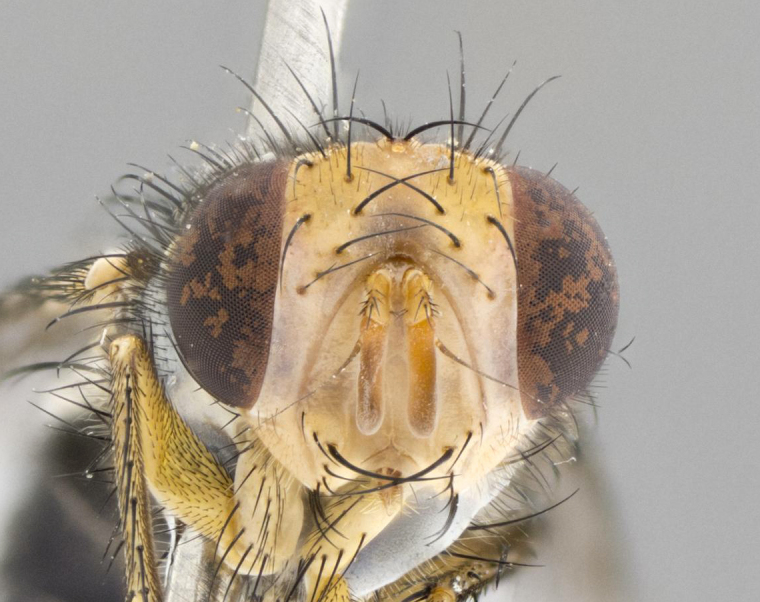
head in frontal view

**Figure 1f.**
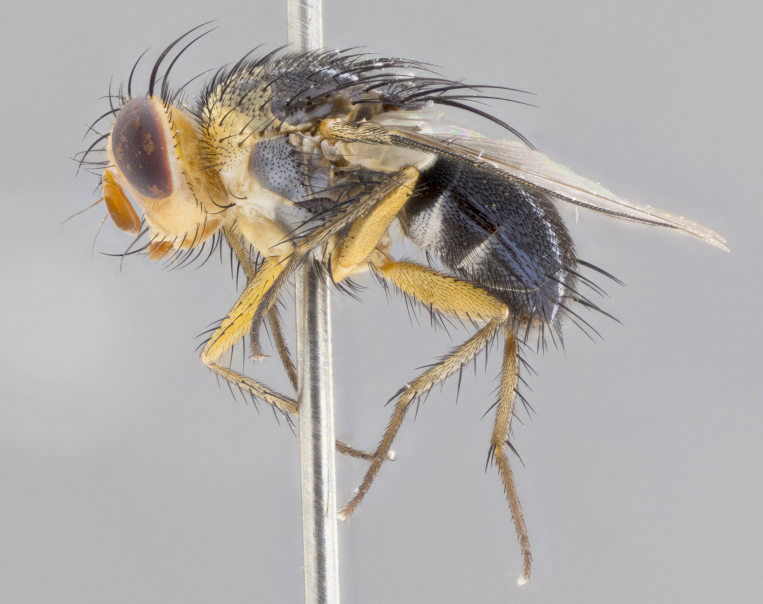
habitus in lateral view

**Figure 2a.**
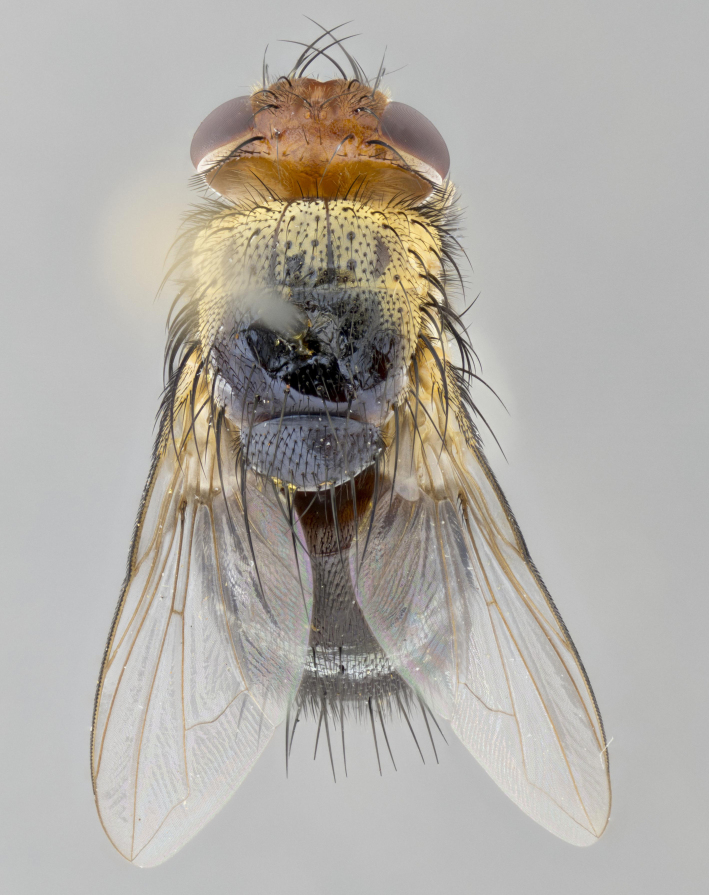
habitus in dorsal view

**Figure 2b.**
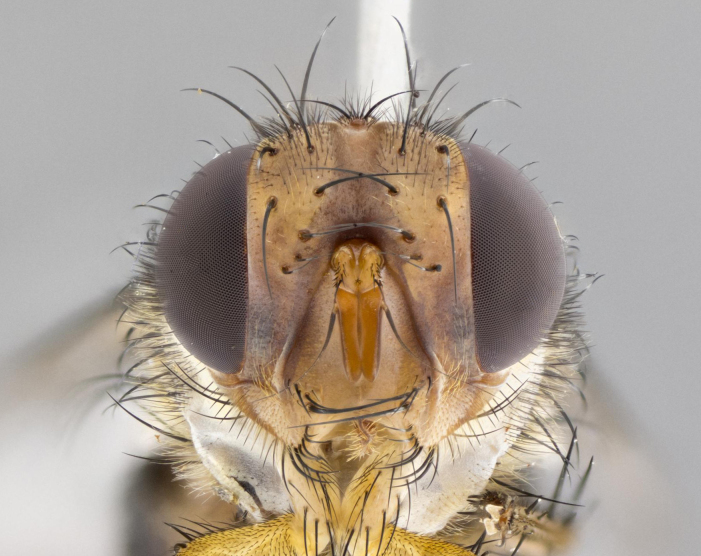
head in frontal view

**Figure 2c.**
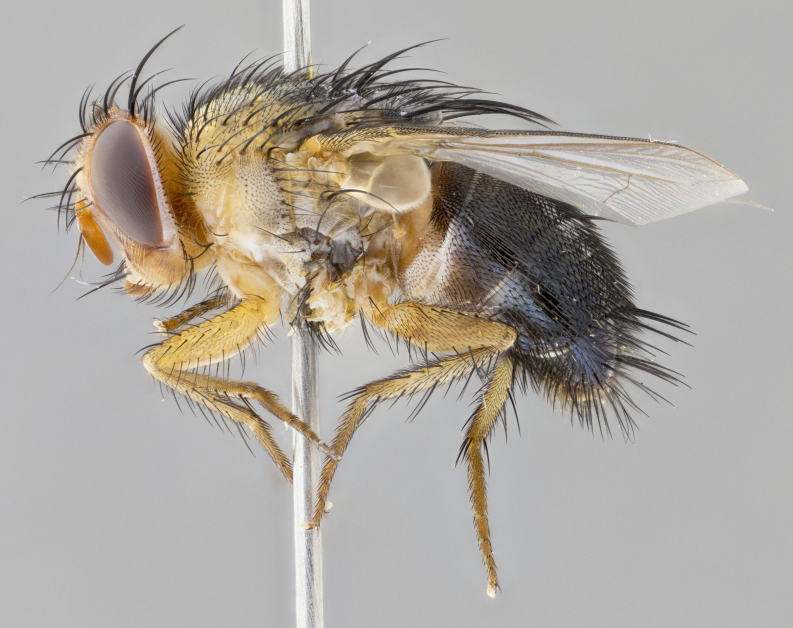
habitus in lateral view

**Figure 2d.**
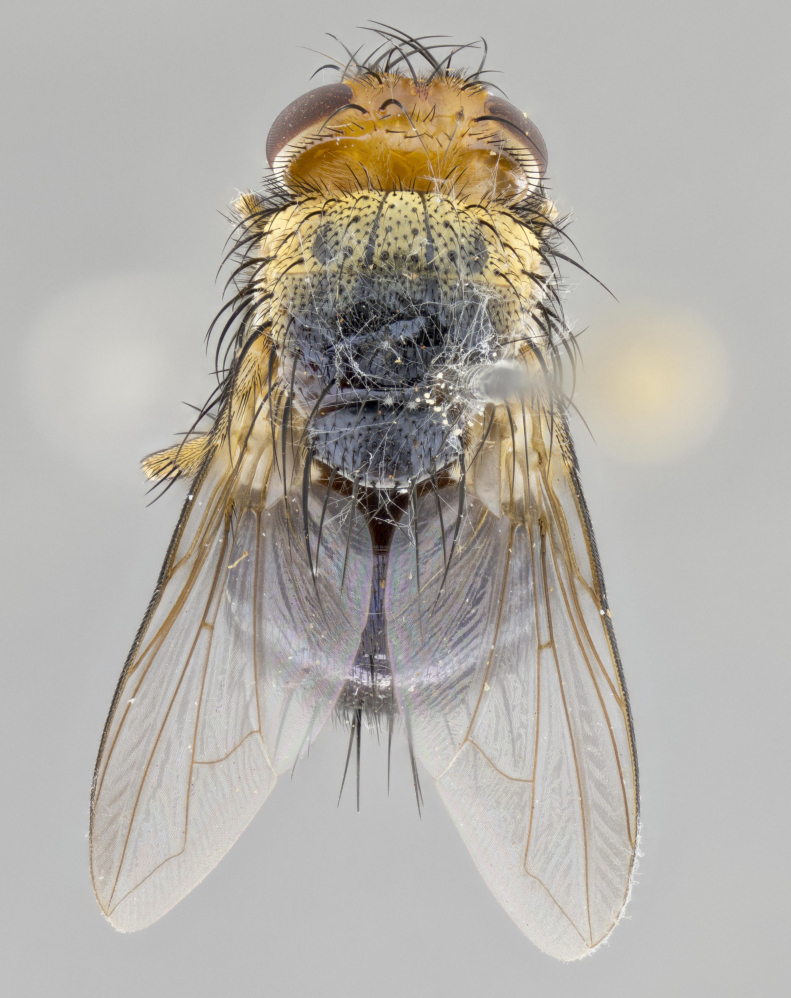
habitus in dorsal view

**Figure 2e.**
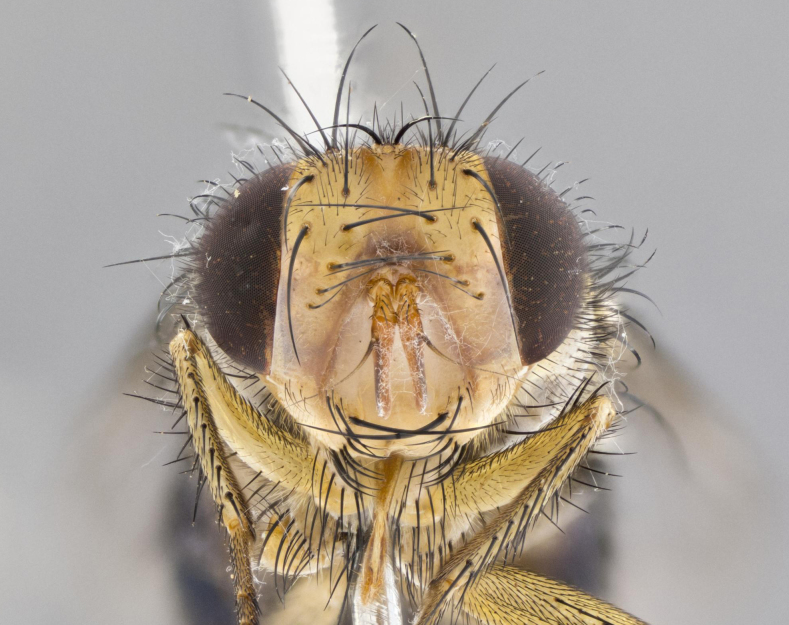
head in frontal view

**Figure 2f.**
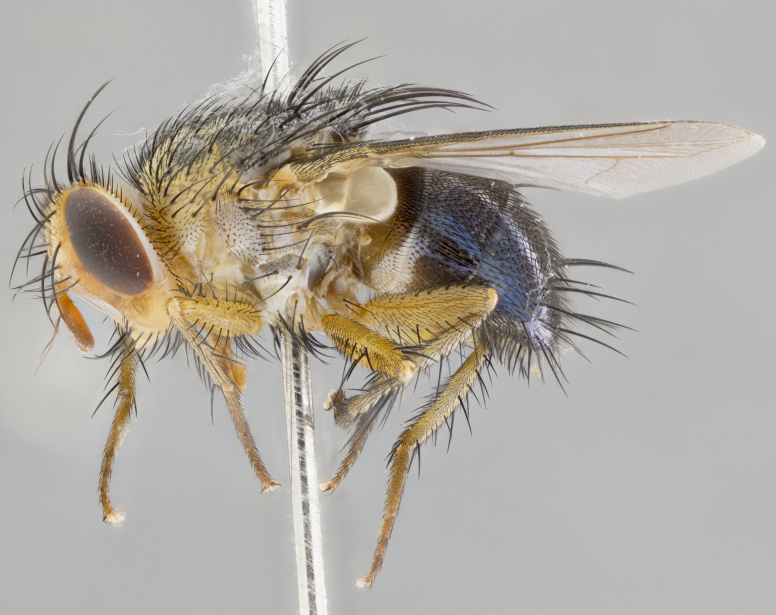
habitus in lateral view

**Figure 3a.**
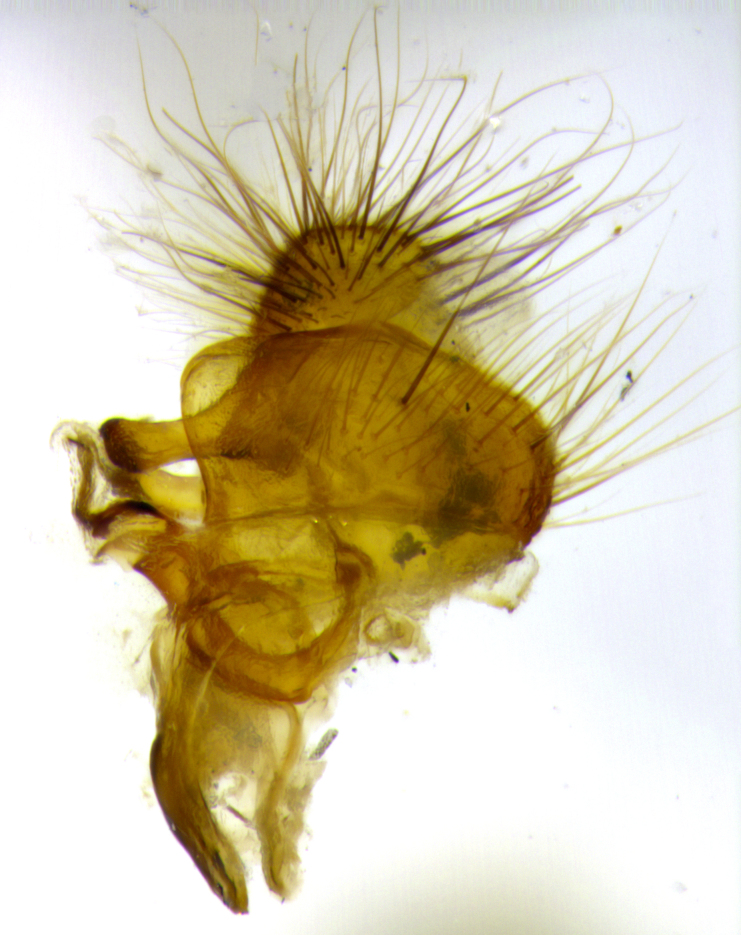
terminalia in lateral view

**Figure 3b.**
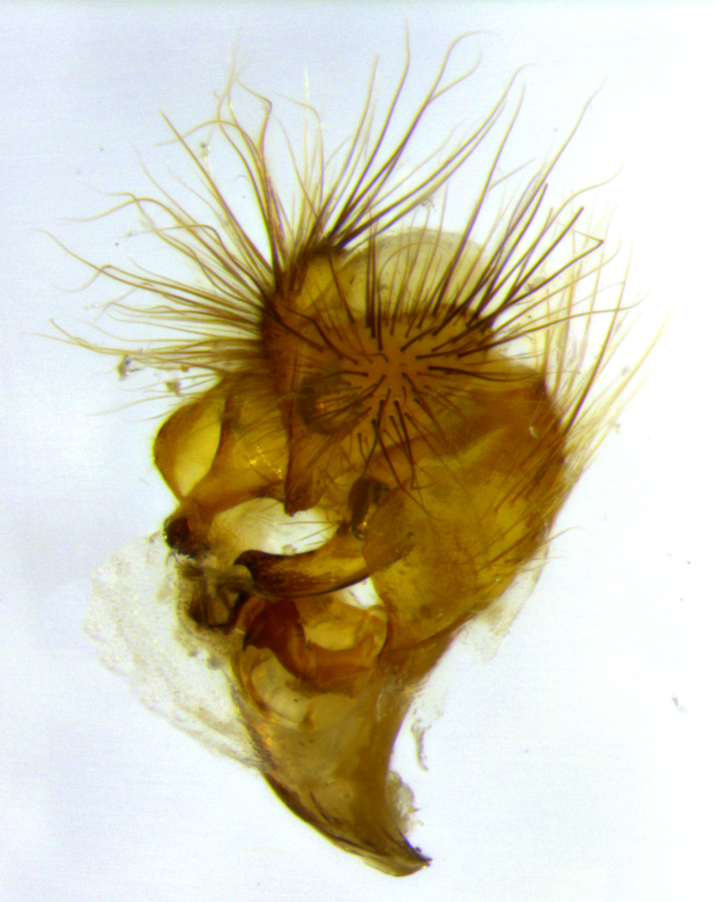
terminalia in oblique fronto-lateral view,showing orientation of surstylus relative to cercus

**Figure 3c.**
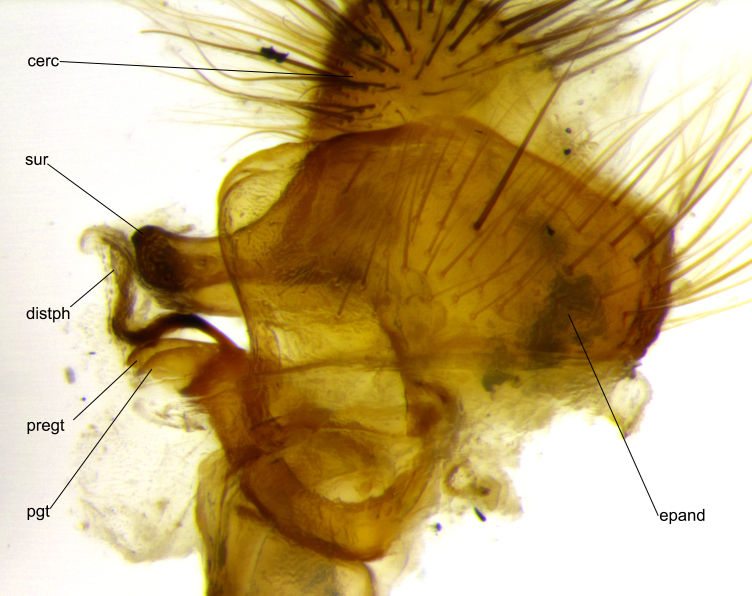
phallus in lateral view

**Figure 3d.**
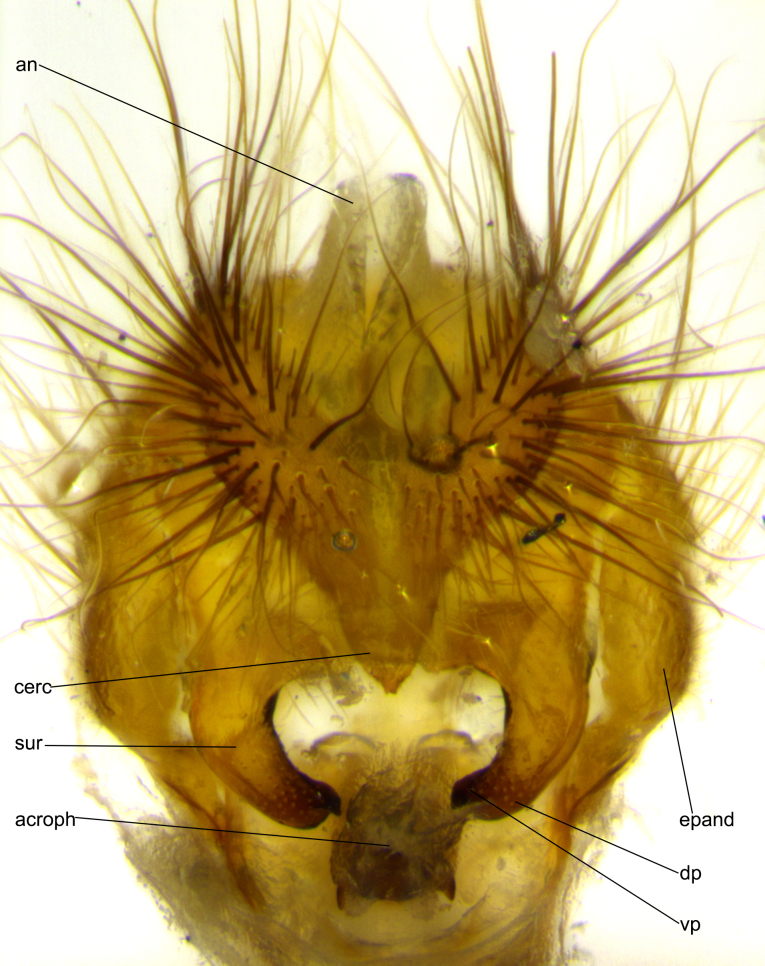
terminalia in posterior view

**Figure 3e.**
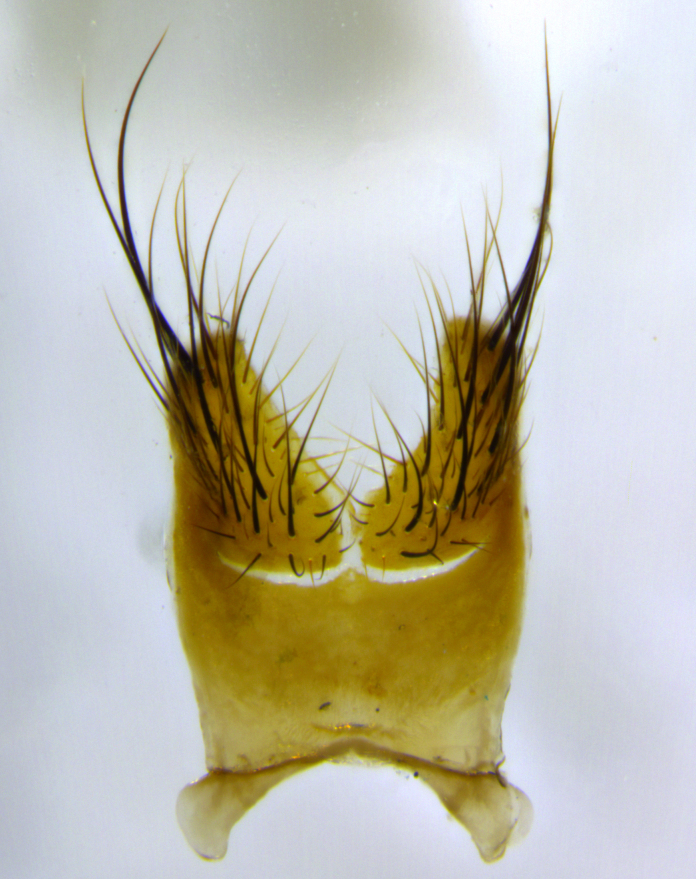
sternite 5 in ventral view
