# Supplementary material for: A new species of Phosocephala Townsend, 1908 (Diptera: Tachinidae) from Area de Conservación Guanacaste in northwestern Costa Rica
Source: Biodivers Data J. 2016 Apr 25;(4):e7863. doi: 10.3897/BDJ.4.e7863 (PMC4867709; doi:10.3897/BDJ.4.e7863)
Supplement: Supplementary material 1 — ACG Phosocephala NJ tree [file biodiversity_data_journal-4-e7863-s001.pdf]

BOLD TaxonID Tree

```
Title           : SEARCH: Tax(Phosocephala) [SEARCH1]
Date            : 15-January-2016
Data Type       : Nucleotide
Distance Model  : Kimura 2 Parameter
Marker          : COI-5P
Codon Positions : 1st, 2nd, 3rd
Labels          : Extra Info, SampleID, ProcessID
Filters         : Length > 200
Colorization    : [blue]=Stop Codons [red]=Contamination or misidentification

Sequence Count  : 5
Species count   : 2
Genus count     : 1
Family count    : 1
Unidentified    : 0
```

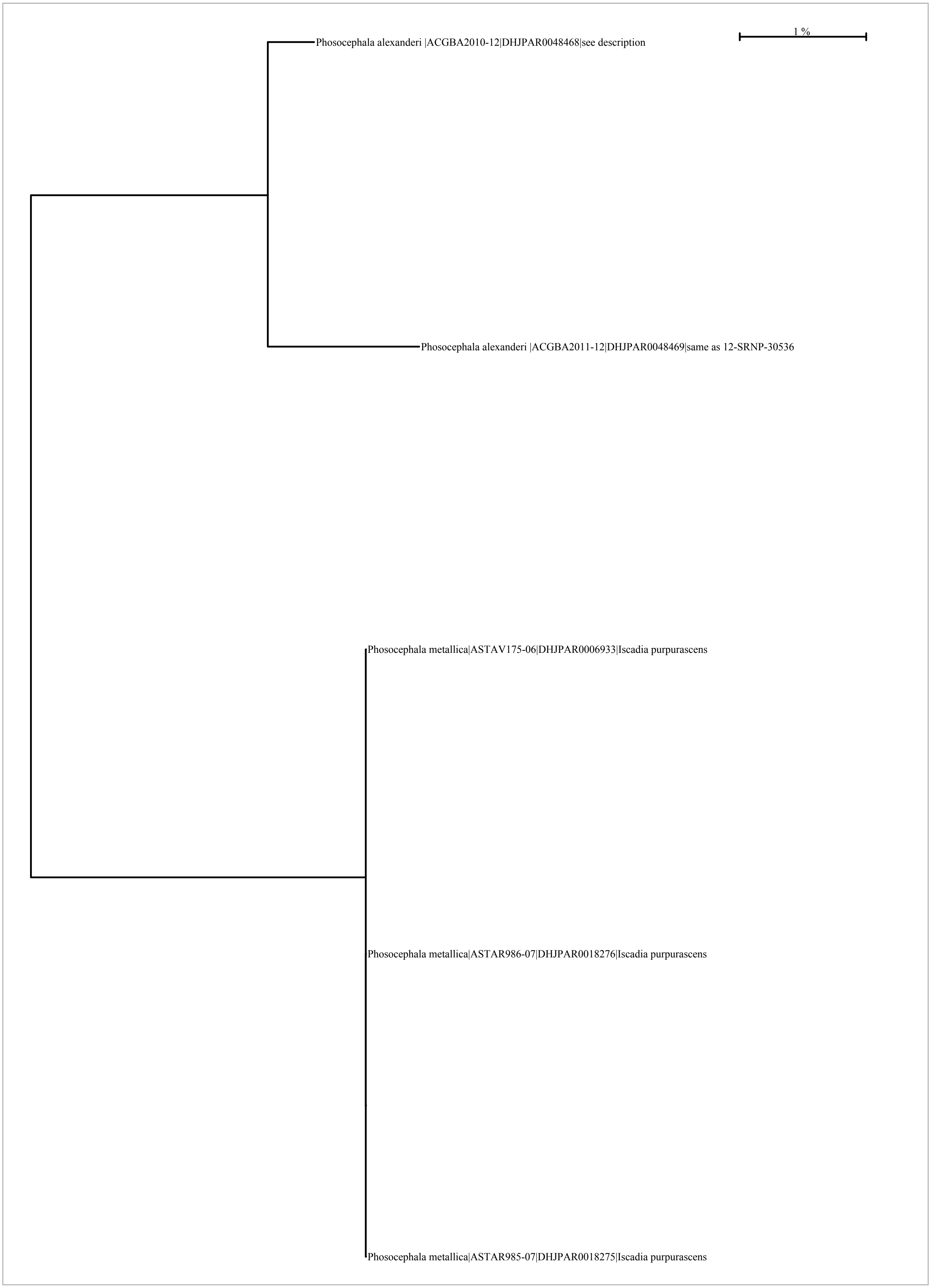
1 %
Phosocephala alexanderi |ACGBA2010-12|DHJPAR0048468|see description
Phosocephala alexanderi |ACGBA2011-12|DHJPAR0048469|same as 12-SRNP-30536
Phosocephala metallica|ASTAV175-06|DHJPAR0006933|Iscadia purpurascens
Phosocephala metallica|ASTAR986-07|DHJPAR0018276|Iscadia purpurascens
Phosocephala metallica|ASTAR985-07|DHJPAR0018275|Iscadia purpurascens
